# Molecular Characterization of Tick-Borne Pathogens in Jiangxi Province: A High Prevalence of *Rickettsia*, *Anaplasma* and *Ehrlichia* in *Rhipicephalus microplus* in Cattle from Ganzhou City, China

**DOI:** 10.3390/pathogens14080770

**Published:** 2025-08-04

**Authors:** Jia He, Meng Yang, Zhongqiu Teng, Peng Wang, Junrong Liang, Yusheng Zou, Wen Wang, Na Zhao, Tian Qin

**Affiliations:** 1 National Key Laboratory of Intelligent Tracking and Forecasting for Infectious Diseases, National Institute for Communicable Disease Control and Prevention, Chinese Center for Disease Control and Prevention, Beijing 102206, China; hejia@icdc.cn (J.H.); tengzhongqiu@icdc.cn (Z.T.); liangjunrong@icdc.cn (J.L.); wangwen@icdc.cn (W.W.); zhaona@icdc.cn (N.Z.); 2 Jiangxi Provincial Center for Disease Control and Prevention, Nanchang 330029, China; ymss921@126.com (M.Y.); 350943234@163.com (P.W.); 3 Yudu County Center for Disease Control and Prevention, Nanchang 330029, China; ydfyz2004@163.com

**Keywords:** tick, Jiangxi, *Rhipicephalus microplus*, tick-borne pathogens

## Abstract

*Rickettsia*, *Anaplasma*, and *Ehrlichia* species are emerging tick-borne pathogens that cause zoonotic diseases, including rickettsiosis, anaplasmosis, and ehrlichiosis in both human and animal populations. This study aimed to investigate the prevalence of these pathogens in cattle-associated ticks from Ganzhou City, Jiangxi Province, China. Through molecular characterization using multilocus sequence analysis (16S *rRNA*, *gltA*, *groEL*, and *ompA* genes), we analyzed 392 *Rhipicephalus microplus* ticks collected from March to September in 2022. The PCR results showed that eight Rickettsiales bacteria were detected, including two species of *Rickettsia* (51/392, 13.0%), four species of *Anaplasma* (52/392, 13.3%), and two species of *Ehrlichia* (70/392, 17.9%). Notably, the circulation of multiple pathogen species within *R. microplus* populations demonstrates significant microbial diversity in this region. Further consideration and investigation should be given to the possible occurrence of rickettsiosis, ehrlichiosis, and anaplasmosis in humans and domestic animals. Our study provides critical baseline data for developing targeted surveillance strategies and informing public health interventions against tick-borne diseases in southeastern China.

## 1. Introduction

Ticks, the second most significant arthropod vectors after mosquitoes, surpass other hematophagous species in their ability to transmit a diverse range of pathogens. They have been documented to carry at least 83 viral species, 31 bacterial species, and 32 protozoan species [1]. Tick bites can cause human diseases, most of which are important natural infectious and zoonotic diseases, such as severe fever with thrombocytopenia syndrome, tick-borne typhus, tick-borne encephalitis, Crimean–Congo hemorrhagic fever, Q fever, Lyme disease, human granulocytic anaplasmosis, and *Bartonella* infection. Annually, tick-borne pathogens cause more than 100,000 cases of human disease globally [2].

*Rhipicephalus microplus*, formerly known as *Boophilus microplus*, is classified as belonging to the family Ixodidae. Its life cycle consists of four stages: egg, larva, nymph, and adult. Each stage requires a blood meal to molt and complete metamorphosis [3]. The *R. microplus* exhibiting a single-host life cycle predominantly infests cattle, earning it the common name “bovine lice” in China. Over the past few decades, it has been widely regarded as the most economically significant ectoparasite affecting cattle worldwide [4], particularly in tropical and subtropical regions [5]. It can carry and transmit a wide range of human and animal pathogens. Some of these pathogens include severe fever with thrombocytopenia syndrome virus, *Babesia bovis*, *Ehrlichia ruminantium*, and *Ehrlichia canis* and the spotted fever group *Rickettsia*, *Anaplasma phagocytophilum*, *Anaplasma marginale*, and *Anaplasma capra*, making this tick an important vector of infectious diseases threatening both human and animal health [6]. Furthermore, about 80% of the world’s cattle population is affected by ticks and tick-borne pathogens, which cause severe economic losses due to the costs associated with parasite control as well as reduced fertility, decreased body weight, and diminished milk production [7,8].

Rickettsiales bacteria, including the spotted fever group of *Rickettsia* (SFGR), *Anaplasma*, and *Ehrlichia*, are recognized as important tick-borne pathogens [9]. Importantly, as urbanization progresses, the risk of human and animal exposure to ticks gradually increases [10]. Thus, it is of the utmost importance to strengthen the surveillance of tick-borne pathogens. However, comprehensive data on the prevalence and diversity of Rickettsiales bacteria, specifically *R. microplus* that are infesting cattle within Ganzhou city, a significant livestock region in southern China, remains largely unknown. This study performed molecular biological detection on ticks collected from cattle in Ganzhou city during the period from March to September 2022, aiming to understand the diversity of Rickettsiales bacteria carried by ticks in this area, providing a scientific basis for the prevention and control of rickettsioses.

## 2. Materials and Methods

### 2.1. Tick Collection and Identification

Ganzhou city, situated in southern Jiangxi Province (latitude 24.48° N–27.15° N, longitude 113.90° E–116.63° E), occupies the southern periphery of the mid-subtropical zone. This subtropical mountainous region features a humid monsoon climate, creating optimal habitats for tick survival and reproduction [11]. Its terrain is mainly composed of mountains, hills, and basins. Livestock often become heavily infested with ticks when they graze in the fields.

**Figure 1:**
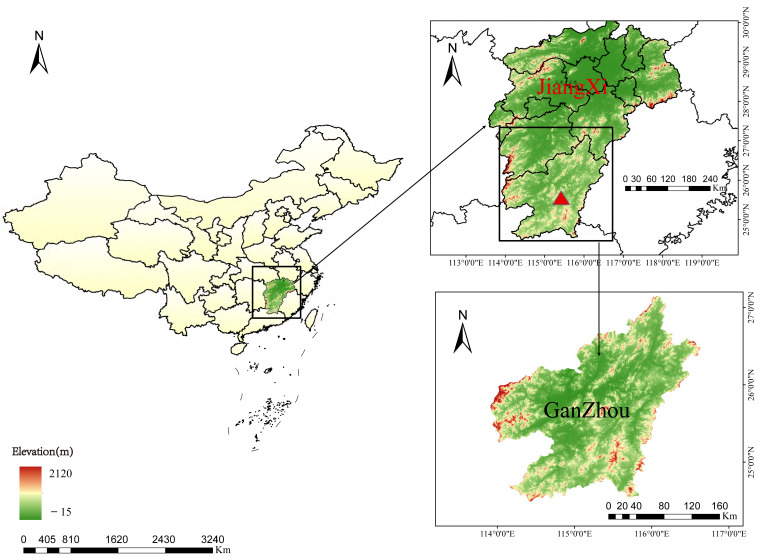
A map showing the sampling region of ticks in Ganzhou city, Jiangxi Province, China (indicated by the red triangle). The map approval number is GS (2024)0650. It was constructed using ArcGIS 10.8 software.

From March to September in 2022, a total of 392 ticks were collected from 30 cattle in Ganzhou, Jiangxi Province (Figure 1). This included 8 yellow cattle and 22 dairy cows. The sampling location map (Figure 1) was generated using ArcGIS 10.8 software (Environmental Systems Research Institute, Redlands, CA, USA). The underlying elevation data were sourced from the Geospatial Data Cloud platform of the Computer Network Information Center, Chinese Academy of Sciences (Geospatial Data Cloud, CAS), specifically the GDEMV3 30M resolution Digital Elevation Model (DEM) data. This map was reviewed and approved by the competent national authorities with the approval number GS (2024) 0650.

All ticks were first identified by trained technicians using an optical microscope based on the differences in their head structure and body shape [12]. The tick species were further confirmed by PCR amplification of the cytochrome c oxidase subunit 1 (COI) gene [13]. All ticks were stored at −80 °C prior to DNA extraction.

### 2.2. DNA Extraction

First, the ticks were washed for 10–15 min, respectively, with 0.1% parathion, 75% ethanol, and Sucrose-Phosphate-Glutamate (SPG) [14], with shaking during the process. After air-drying, they were placed in 2 mL centrifuge tubes with steel beads, and a 200 μL SPG solution was added. Then, homogenization was achieved using a grinding instrument (Retsch MM 400, Haan, Germany) with the following conditions: frequency 30Hz and time 2 min. Finally, genomic DNA was extracted using the QIAamp DNA Mini Kit (QIAGEN, Cat No. 51304, Hilden, Germany) according to the manufacturer’s protocol. The DNA was then eluted in 200 µL RNase-free water for subsequent analysis. All DNA samples were stored at −20 °C. Note that the PCR detection of pathogens in engorged ticks cannot distinguish whether the detected pathogen DNA originated from an infected tick or from residual bovine blood in the tick’s gut.

### 2.3. Polymerase Chain Reaction

Nested and semi-nested PCR were used to detect pathogens, namely, *Rickettsia*, *Anaplasma*, and *Ehrlichia*. *Rickettsia* spp. were detected by nested PCR, as described previously using primers targeting a conserved region of the 16S *rRNA* gene, resulting in a PCR product of approximately 900 bp [15]. The DNA samples were also screened for Anaplasmataceae bacteria as described previously, amplifying a 500 bp fragment of the 16S *rRNA* gene [16]. The primers were custom-synthesized by Beijing DIA-UP Biotechnology Co., LTD. (Beijing, China). The target fragment was verified by agar-gel electrophoresis and further confirmed by Sanger sequencing at Tianyi Huiyuan Biotechnology Company (Beijing, China). The 16S *rRNA* sequences were analyzed using BLASTn v2.16.0+ (MegaBLAST optimization for highly similar sequences) against the NCBI Core Nucleotide Database (core_nt release 2024_05) for species identification. To precisely determine the bacterial species and analyze the genetic diversity of detected strains, nearly complete 16S *rRNA* gene sequences (1000 bp) and partial sequences of *gltA* (961 bp), *groEL*(1021bp), and *ompA* (700 bp) were obtained for representative *Rickettsia* strains using previously described primers [16]. For *Anaplasma* and *Ehrlichia*, the 16S *rRNA*, *gltA*, and *groEL* genes were amplified using nested primers [15,16,17,18,19,20,21]. All PCR-positive amplicons were subsequently sent to Beijing Tianyi Huiyuan Biotechnology Co., Ltd. for paired-end sequencing. All sequences were uploaded to the GenBank Database, and the accession numbers are shown in Table S1. All the primers involved in this study are listed in Table S2.

### 2.4. Phylogenetic Analysis

The sequences were edited and assembled using the SeqMan software (DNASTAR, Madison, WI, USA; SeqMan Pro 12.1.0). Sequence comparisons were conducted against the NCBI GenBank nucleotide databases (core_nt). Additionally, the neighbor-joining (NJ) method was adopted for multiple sequence alignments, and a phylogenetic tree was constructed in MEGA 7.0. To evaluate the reliability of the results, 1000 bootstrap replications were carried out. All PCR-positive samples were sequenced for target genes. For concise visualization, only representative sequences are shown in phylogenetic trees. When multiple target genes are used for detection, as long as two or more independent gene fragments are successfully amplified and sequenced to confirm specific sequences, it can be clearly determined that the sample is positive.

## 3. Results

### 3.1. Tick Identification

**Figure 2:**
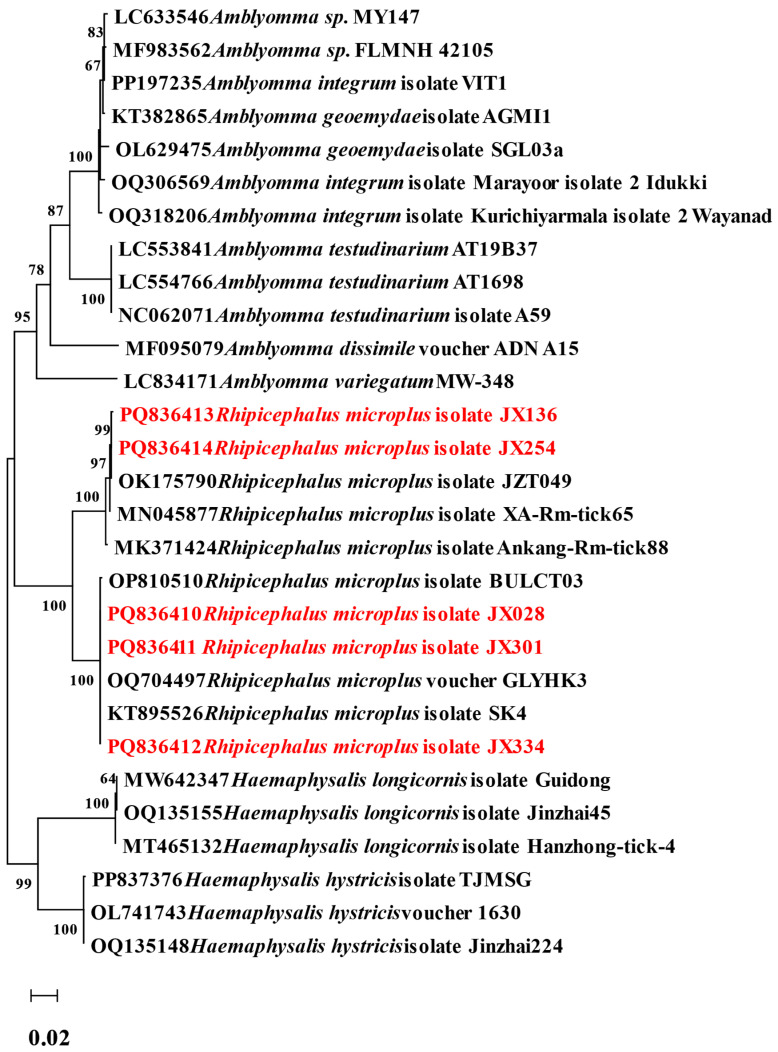
The phylogenetic analysis of ticks was conducted based on the nucleotide sequences of the COI gene. The resulting tree shows the relationship of *R. microplus* to other tick species. Sequences obtained in this study are marked in red.

A total of 392 ticks were collected in Ganzhou city, Jiangxi Province, from March to September 2022, including 97 nymphs (24.7%) and 295 adults (75.3%). Engorgement analysis indicated 257 semi-engorged (65.6%) and 135 fully engorged specimens (34.4%) (Table S3). All of them were collected from the body surface of cattle, including 8 yellow cattle and 22 dairy cows. All 392 ticks underwent morphological and molecular identification based on the COI gene. They belonged to the *Rhipicephalus microplus* (100%, 392/392). The phylogenetic tree constructed based on representative COI gene sequences (selected from the fully sequenced cohort) is shown in Figure 2. The *R. microplus* isolates JX136 and JX354 showed 100% identity (Query cover: 100%, E-value: 0.0) with previously reported *R. microplus* (OK175790). The *R. microplus* isolates JX028, JX301, and JX334 clustered within the same branch in the phylogenetic tree (Query cover: 100%, E-value: 0.0). From the 392 ticks subjected to COI gene sequencing, five representative COI gene sequences were selected and submitted to GenBank, with accession numbers PQ836410-PQ836414.

### 3.2. Rickettsia Bacteria Detected in Ticks

**Figure 3:**
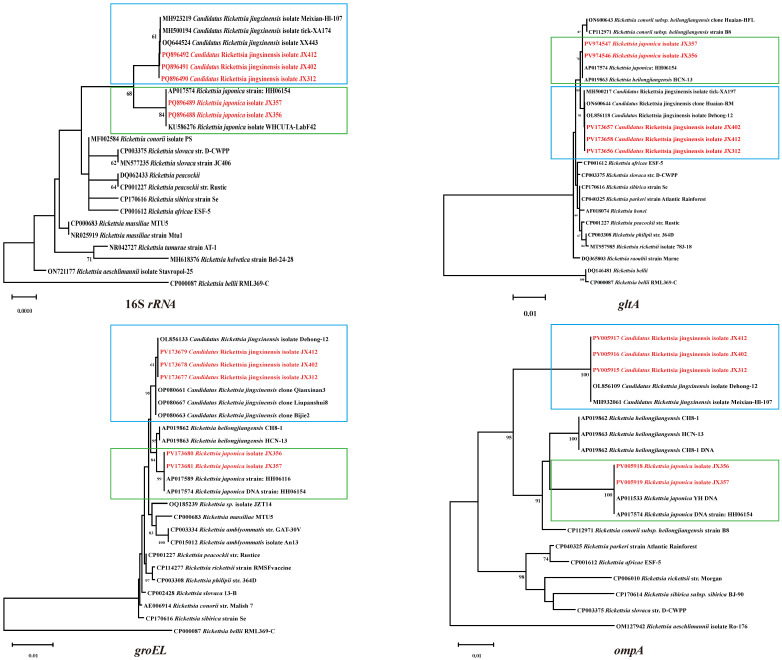
Phylogenetic trees were constructed based on the nucleotide sequences of the 16S *rRNA*, *gltA*, *groEL*, and *ompA* genes of *Rickettsia* strains. Sequences obtained in this study are marked in red. Blue: *Candidatus* R. jingxinensis. Green: *R. japonica*.

To gain a better understanding of the phylogenetic relationships between the *Rickettsia* spp. in this study and those described previously, nucleotide alignments and analysis of *Rickettsia* spp. were carried out based on the 16S *rRNA*, *gltA*, *groEL*, and *ompA* genes. In the phylogenetic tree, these genes clustered together with the corresponding genes of *Candidatus* Rickettsia jingxinensis and *Rickettsia japonica*, accompanied by high homology (Figure 3). In the 16S *rRNA* phylogenetic tree, the *Ca. R. jingxinensis* isolates JX312, JX402, and JX412 in the blue box exhibited 100% homology to the same branch as *Ca. R. jingxinesis*. Similarly, *R. japonica* isolates JX356 and JX357 in the green boxes were clustered with known *R. japonica* strains. The sequences of *gltA*, *groEL*, and *ompA* for *Ca. R. jingxinensis* were identical to those of the *Ca. R. jingxinensis* isolate Dehong (OL856118-120). The other three genes (*gltA*, *groEL*, and *ompA* gene fragments) of *R. japonica* showed high homology with the *R. japonica* strain HH06154 (query cover: 100%, E-value: 0.0). Based on the comprehensive analysis of four genes, it was determined that two *Rickettsia* species were identified in this tick species, including *Ca. R. jingxinensis* and *R. japonica*. Their positive rates were 3.8% (15/392) and 9.2% (36/392), respectively (Table S4).

### 3.3. Anaplasma Bacteria Detected in Ticks

**Figure 4:**
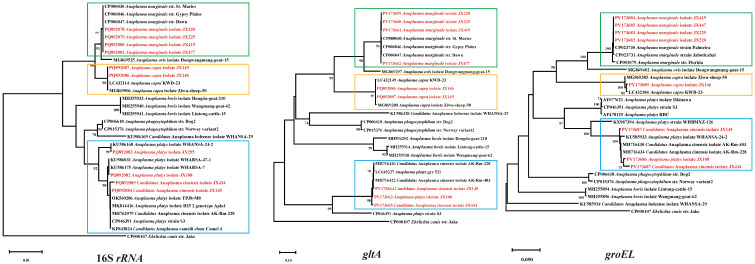
Phylogenetic trees were constructed based on the nucleotide sequences of the 16S *rRNA*, *gltA*, and *groEL* genes of *Anaplasma* strains. Sequences obtained in this study are marked in red. Blue: Co-clustering of *A. platys* and *Candidatus* A. cinensis isolates. Green: *Anaplasma marginale*. Yellow: *Anaplasma capra*.

Four *Anaplasma* species were identified in the ticks with a combined positive rate of 13.3% (52/392): *A. platys* (0.5%, 2/392), *Ca. A. cinensis* (0.5%, 2/392), *A. marginale* (11.7%, 46/392), and *A. capra* (0.5%, 2/392). *Anaplasma platys* detected in the current study was classified into two genotypes in the phylogenetic tree based on the 16S *rRNA* genes. Isolates JX108 and JX295 were most closely related to strains WHARSA-47-1 and WHARSA-24-2, respectively. The sequences of the three genes shared 99.46–100% identity with *A. platys* strains from other provinces of China. In the phylogenetic tree constructed based on 16S *rRNA*, *gltA*, and *groEL* genes, although JX145 and JX434 clustered with both *A. platys* and *Ca. A. cinensis*, a detailed analysis revealed that the sequences of all three genes clustered with the *Ca. A. cinensis* isolate AK-Rm-228 (MH762079, MH716426, MH716434), leading to their identification as *Ca. A. cinensis*. *Anaplasma marginale*, a widely distributed animal pathogen, shared 99.87–100% homology with other *A. marginale* strains on the same branch of the phylogenetic tree. Its prevalence was the highest among the *Anaplasma* species detected, reaching 11.7%, which accounted for 88% of all *Anaplasma* positive samples. One *A.capra* isolate was closest to *A. capra* KWD-23 (LC432184), with 99.85–100% identity based on 16S *rRNA*, *gltA*, and *groEL* (Figure 4).

### 3.4. Ehrlichia Bacteria Detected in Ticks

**Figure 5:**
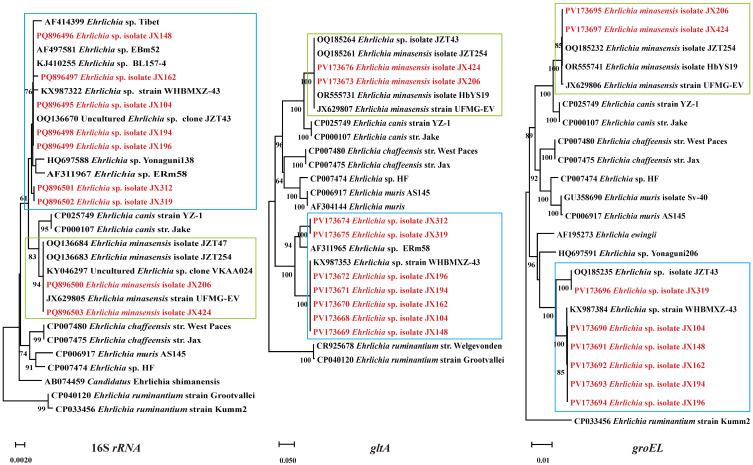
Phylogenetic trees were constructed based on the nucleotide sequences of the 16S *rRNA*, *gltA*, and *groEL* genes of *Ehrlichia* strains. Sequences obtained in this study are marked in red. Blue: *Ehrlichia* sp. Green: *Ehrlichia minasensis*.

Of the 392 ticks screened in Ganzhou city, 70 (17.9%) tested positive for *Ehrlichia*, including two species: *E. minasensis* (6/392, 1.5%) and *Ehrlichia* spp. (64/392, 16.3%). The 16S *rRNA*, *gltA*, and *groEL* sequences of *E. minasensis* isolates JX206 and JX424 showed 100% identity with the *E. minasensis* isolate JZT254 (from Jinzhai County, Anhui Province, China). The 16S *rRNA* sequences of representative *Ehrlichia* sp. strains had three different nucleotides, resulting in their division into distinct clades in the phylogenetic tree. The clade containing the *Ehrlichia* sp. isolate JX104 exhibited 99.82–100% homology with other *Ehrlichia* in the same branch. Its 16S *rRNA*, *gltA*, and *groEL* sequences showed 99.1%, 100%, and 100% homology to the *Ehrlichia* sp. strain WHBMXZ-43, respectively. The 16S *rRNA* gene sequences of the other group of *Ehrlichia* sp. (*Ehrlichia* sp. isolate JX312 and JX319) shared 99.91% and 99.65% homology with *Ehrlichia* sp. BL157-4 and *Ehrlichia* sp. ERm58, respectively. The *gltA* sequences were closely related to *Ehrlichia* sp. ERm58 with 94.50% homology. The *groEL* gene sequences of the *Ehrlichia* sp. isolate JX319 were 100% identical to those of the *Ehrlichia* sp. isolate JZT43 (Figure 5).

### 3.5. Co-Infection of Rickettsiales in Ticks

As shown in Table 1, co-infection with two Rickettsiales pathogens was detected in 13 (3.3%, 13/392) individual ticks. Four (1.0%, 4/392) ticks were co-infected with *Ca. R. jingxinensis* and *Ehrlichia* spp., four (1.0%, 4/392) ticks were co-infected with *R. japonica* and *Ehrlichia* spp., two (0.5%, 2/392) ticks were co-infected with *R. japonica* and *A. marginale*, and one tick was co-infected with *Ca. R. jingxinensis* and *A. marginale*. One tick was co-infected with *Ca. R. jingxinensis* and *A. platys*, and one tick was co-infected with *Ca. R. jingxinensis* and *E. minasensis.*

## 4. Discussion

*Rhipicephalus microplus* is distributed in Asia, Latin America, the Middle East, and East and South Africa [22]. It has been found that *R. microplus* is reported and recorded in a total of 56 countries worldwide, of which 463 coordinates have been reported in China [23]. Studies have shown that there are 18 species of ticks in Jiangxi Province, which belong to two families and six genera [24]. Despite its importance as a vector, the study on *R. microplus* in Ganzhou City, Jiangxi Province, remains limited, and its pathogen spectrum needs to be further studied. To address this knowledge gap, our molecular survey revealed that *R. microplus* in Ganzhou carries a diverse community of Rickettsiales bacteria, specifically detecting two *Rickettsia* (*R. japonica* and *Ca. R. jingxinensis*), four *Anaplasma* (*A. platys*, *A. marginale*, *A. capra*, and *Ca. A. cinensis*), and two *Ehrlichia* species (*E. minasensis* and *Ehrlichia* spp.). This diversity underscores the significant vector potential of this tick in the region.

Crucially, assessing the vector competence of *R. microplus* for the detected pathogens is essential for understanding the transmission risk. *R. microplus* is a well-established vector for *A. marginale* [25], which is the causative agent of bovine anaplasmosis. While infections with *A. marginale* can be fatal in adult cattle, many animals remain asymptomatic despite harboring this pathogen [26]. However, the vector competence of *R. microplus* for the other detected pathogens is less clear or largely unknown. For *Rickettsia japonica*, while several tick species are recognized as primary vectors [27], *R. microplus* has been found infected with *R. japonica* or closely related to SFGR in field surveys in Asia, suggesting that it may play a role in maintenance or potentially act as a secondary vector. *Candidatus* Rickettsia jingxinensis has also been detected in various tick species, including *R. microplus* in China [28,29,30], but its specific vector competence requires further experimental validation. Concerning the *Ehrlichia* species, *Ehrlichia minasensis* and *Ehrlichia* spp. have been detected in *R. microplus* ticks in multiple countries [31,32,33], but definitive proof of *R. microplus* as a competent vector is still lacking.

The detection of *Rickettsia* in ticks has important implications not only for identifying infected ticks, but also for assessing the risk of transmission to humans [34]. In the present study, we detected *R. japonica* (9.2%) and *Ca. R. jingxinensis* (3.8%). The detection of *R. japonica* is a particular public health concern as the causative agent of Japanese spotted fever (JSF); it is transmitted primarily through tick bites and was first diagnosed in Japan in 1984 [35]. In recent years, the geographical distribution of *R. japonica* in ticks in China has been expanding, which indicates an increased risk to human health. Moreover, delayed diagnosis and treatment of JSF can be fatal [36]. In this study, we obtained 16S *rRNA*, *groEL*, and *ompA* gene fragments of *R. japonica*, which were 100% homologous to the existing gene fragments in the database, revealing the potential risk of JSF to local humans. *Candidatus* Rickettsia jingxinensis, named for its geographical origin, is widespread in China [37]. The *Ca. R. jingxinensis* variant *Rickettsia* sp. XY118 has been found in tick-bitten patients, suggesting its potential as a human pathogen and underscoring the need for human infection monitoring [38]. Its detection in Ganzhou ticks necessitates heightened awareness and surveillance for potential human infections in this area.

Anaplasmosis is a priority disease in the Terrestrial Animal Health Code of the World Organization for Animal Health. In the present study, four bacterial species belonging to the genus *Anaplasma* were identified: *A. platys*, *A. marginale*, *A. capra*, and *Ca. A. cinensis*. *Anaplasma platys* was first described as a canine pathogen that infects the host’s platelets, and was identified as the etiological agent of canine cyclic thrombocytopenia, affecting cats and ruminants, including cattle, goats, camels, buffalo, and red deer [39]. Although the *R. sanguineus* tick is thought to be the primary vector of *A. platys* transmission, its DNA has also been detected in other tick species. In 2013, the first human case was reported when *A. platys* DNA was detected in a female veterinarian’s blood sample [40]. In 2014, another study reported two case reports of *A. platys* detection in two women from Venezuela [41]. Our finding of *A. platys* in cattle ticks suggests a potential, albeit possibly secondary, route for human exposure in this agricultural setting. *Anaplasma marginale* is widely distributed throughout the world and is an important animal pathogen that can cause bovine anaplasmosis, which is characterized by fever, anemia, jaundice, abortion, wasting, and other symptoms; transplacental transmission has also been reported [42,43,44]. Notably, *A. marginale* was detected at a high prevalence of 11.7%. This high prevalence is alarming for animal health given its role as the primary agent of bovine anaplasmosis, causing significant economic losses through morbidity and mortality. Its presence poses a direct and substantial threat to the local cattle industry in Ganzhou. *Anaplasma capra* has attracted widespread attention since it was first identified in goats and patients in China in 2015 [45]. Modeling predicts that this zoonotic pathogen could spread to wider areas; as such, its implications for public health and veterinary safety warrant further study [46]. *Candidatus* Anaplasma cinensis is a novel unclassified *Anaplasma* species genetically related to *A. platys*, which was first identified in ticks in the southeastern Shaanxi Province [47]. Given its phylogenetic relation to *A. platys* and the zoonotic potential emerging within the Anaplasma genus, its epidemiology and potential impact on human and animal health warrant focused investigation.

The genus *Ehrlichia* belongs to the family Anaplasmataceae and consists of six recognized species: *E. canis*, *E. chaffeensis*, *E. muris*, *E. ewingii*, *E. ruminantium*, and *E. minasensis* [48,49]. *Ehrlichia minasensis*, an *Ehrlichia* closely related to *E. canis*, was initially reported in Canada and Brazil [50,51]. This bacterium has now been reported in Pakistan, Malaysia, China, Ethiopia, South Africa, and the Mediterranean island of Corsica, suggesting that *E. minasensis* has a wide geographical distribution [52]. In this study, the positive rate of *E. minasensis* was 1.5%, suggesting that *E. minasensis* is circulating among cattle in Ganzhou city. Seroprevalence studies in local cattle are needed to determine the infection prevalence of *E. minasensis* in infected animals. In the present study, uncultured strains belonging to two *Ehrlichia* spp. (*Ehrlichia* sp. isolates JX104, JX148, JX162, JX194, and JX196 and *Ehrlichia* sp. isolates JX312 and JX319) were detected in this study. *Ehrlichia* sp. isolates JX104, JX148, JX162, JX194, and JX196 are closely related to the *Ehrlichia* sp. strain WHBMXZ-43 identified in *R. microplus* from Wuhan, China. *Ehrlichia* sp. isolates JX312, and JX319 were clustered together with *Ehrlichia* sp. EmR58 is found in African ticks. Although clinical disease in humans has not yet been definitively linked to these specific strains, their presence in ticks feeding on cattle represents a potential zoonotic hazard. Epidemiological evidence increasingly shows that microbes initially considered non-pathogenic can later cause human disease. The genetic divergence of these Ehrlichia spp. isolates underscores the diversity of potentially infectious agents carried by *R. microplus* in Ganzhou and highlights a critical gap in our understanding of their pathogenicity. A proactive assessment of their virulent potential is imperative. Such comprehensive surveillance is crucial for public health preparedness, given the accelerated geographic expansion of tick populations and their associated microbial communities under current climate change scenarios.

*Rickettsia*, *Anaplasma*, and *Ehrlichia* were detected in *R. microplus* within the study area, indicating significant zoonotic risks to livestock workers and farmers, especially in rural areas (Table S4). In addition, these pathogens may also cause economic losses and food security issues through livestock morbidity and mortality. Therefore, understanding the key aspects of these bacteria, including their ecology, genetic diversity, and pathogenicity, is essential for developing targeted risk mitigation strategies locally.

Several limitations should be acknowledged in our study. First of all, only a single host species was included in the sample; therefore, it is necessary to expand the host species in subsequent studies. Second, due to the absence of host blood data, this inhibited our ability to conclusively determine the immediate source of the pathogen within the detected ticks (host blood vs. prior tick infection). Consequently, collecting host blood will also be necessary for our subsequent work. Third, as noted in the Methods section, our PCR-based approach on engorged ticks cannot differentiate the source of pathogen DNA. Thus, further investigations in cattle and free ticks are required to clarify transmission cycles. Meanwhile, pathogen cultivation is part of our ongoing work.

## 5. Conclusions

In summary, our study revealed a diversity of pathogenic Rickettsial species in *R. microplus* ticks from Jiangxi Province, indicating potential threats to human and animal health in China. Our results may contribute to the current understanding of the biodiversity of Rickettsiales bacteria circulating in this region.

## Figures and Tables

**Table 1:** Coinfection of *Rickettsia*, *Anaplasma*, and *Ehrlichia* in ticks in Ganzhou city, China.

Ticks	Coinfection			Number
	*Rickettsia*	*Anaplasma*	*Ehrlichia*	
*Rhipicephalus microplus*	*R. japonica*	*A. marginale*		2
	*Ca. R. jingxinensis*	*A. marginale*		1
	*Ca. R. jingxinensis*	*A. platys*		1
	*R. japonica*		*Ehrlichia* sp.	4
	*Ca. R. jingxinensis*		*Ehrlichia* sp.	4
	*Ca. R. jingxinensis*		*E. minasensis*	1

## Data Availability

All the sequence files are available from the NCBI database (accession numbers are shown in Table S1).

## Supplementary Materials

The following supporting information can be downloaded at https://www.mdpi.com/article/10.3390/pathogens14080770/s1. Table S1. GenBank numbers of sequences obtained in this study; Table S2. Primers for the amplification of sequences of ticks and tick-borne pathogens; Table S3. Statistical table of developmental stage and blood-soaked state of ticks; Table S4. The positive rate and quantity of Rickettsiales bacteria detected in this study.
