# Supplementary material for: Molecular Characterization of Tick-Borne Pathogens in Jiangxi Province: A High Prevalence of Rickettsia, Anaplasma and Ehrlichia in Rhipicephalus microplus in Cattle from Ganzhou City, China
Source: Pathogens. 2025 Aug 4;14(8):770. doi: 10.3390/pathogens14080770 (PMC12389721; doi:10.3390/pathogens14080770)
Supplement: Supplementary file 1 [file pathogens-14-00770-s001.zip › pathogens-3736706-supplementary.pdf]

Table S1. GenBank numbers of sequences obtained in this study.

	COI	16S *rRNA*	*gltA*	*groEL*	*ompA*
Rhipicephalus microplus isolate JX028	PQ836410	-	-	-	-
Rhipicephalus microplus isolate JX301	PQ836411	-	-	-	-
Rhipicephalus microplus isolate JX334	PQ836412	-	-	-	-
Rhipicephalus microplus isolate JX136	PQ836413	-	-	-	-
Rhipicephalus microplus isolate JX254	PQ836414	-	-	-	-
Rickettsia japonica isolate JX356	-	PQ896488	PV974546	PV173680	PV005918
Rickettsia japonica isolate JX357	-	PQ896489	PV974547	PV173681	PV005919
Candidatus Rickettsia jingxinensis isolate JX312	-	PQ896490	PV173656	PV173677	PV005915
Candidatus Rickettsia jingxinensis isolate JX402	-	PQ896491	PV173657	PV173678	PV005916
Candidatus Rickettsia jingxinensis isolate JX412	-	PQ896492	PV173658	PV173679	PV005917
Anaplasma marginale isolate JX220	-	PQ892078	PV173659	PV173682	-
Anaplasma marginale isolate JX225	-	PQ892079	PV173660	PV173683	-
Anaplasma marginale isolate JX419	-	PQ892080	PV173661	PV173684	-
Anaplasma marginale isolate JX447	-	PQ892081	PV173662	PV173685	-
Anaplasma platys JX108	-	PQ892082	PV173663	PV173686	-
Anaplasma platys JX295	-	PQ892083	-	-	-
Candidatus Anaplasma cinensis isolate JX145	-	PQ892084	PV173664	PV173687	-
Candidatus Anaplasma cinensis isolate JX434	-	PQ892085	PV173665	PV173688	-
Anaplasma capra isolate JX146	-	PQ892086	PV173666	PV173689	-
Anaplasma capra isolate JX149	-	PQ892087	PV173667		-
Ehrlichia sp. isolate JX104	-	PQ896495	PV173668	PV173690	-
Ehrlichia sp. isolate JX148	-	PQ896496	PV173669	PV173691	-
Ehrlichia sp. isolate JX162	-	PQ896497	PV173670	PV173692	-
Ehrlichia sp. isolate JX194	-	PQ896498	PV173671	PV173693	-
Ehrlichia sp. isolate JX196	-	PQ896499	PV173672	PV173694	-
Ehrlichia minasensis isolate JX206	-	PQ896500	PV173673	PV173695	-
Ehrlichia sp. isolate JX312	-	PQ896501	PV173674	-	-
Ehrlichia sp. isolate JX319	-	PQ896502	PV173675	PV173696	-
Ehrlichia minasensis isolate JX424	-	PQ896503	PV173676	PV173697	-

Table S2. Primers for the amplification of sequences of ticks and tick-borne pathogens.

Organism	PCR Method	Gene	Name	Sequences (5′-3′)	Anticipated Amplicon Length	References
Ticks	PCR	*CO* Ⅰ	Tick-*COI*-F	GGTCAACAAATCATAAAGA-TATTGG	700bp	[1]
			Tick-*COI*-R	TAAACTTCAGGGTGAC-CAAAAAATCA		
Rickettsia	semi-nested PCR	16S	R-rrs1-F	GTACGGAATAACTTTTA-GAAAT	900 bp	[2]
			R-rrs1-R1	CATGATGACTTGACRTCGT		
			R-rrs1-R2	CATCTCACGACACGAGCTG		
	semi-nested PCR	16S	R-rrs1-F1	GAAGGCGRTCATYTRGGCT	600 bp	
			R-rrs1-F2	GRTCATYTRGGCTRCAACTG		
			R-rrs1-R	CTGCCTCTTGCGTTAGCT		
	semi-nested PCR	*gltA*	R-*gltA*-F1	CCGGGYTTTATGTCTACTGC	1100 bp	
			R-*gltA*-F2	CTTTATGTCTACTGCKTCTTG		

			R-*gltA*-R	AGCTGTCTWGGTCTGCTGATT		
	semi-nested PCR	*groEL*	R-*groEL*-F1	CCATTACATGATAGAATT-GCAAT	1100 bp	
			R-*groEL*-F2	GAATTGCAATAAAGCC-TATCG		
			R-*groEL*-R	CCATCATTGCTTTTCTTCTATC		
	semi-nested PCR	*ompA*	R-*ompA*-F	TGGCGAATATTTCTCCAAAA	700bp	
			R-*ompA*-R1	AC-CTACATTATCAAHGCCTGT		
			R-*ompA*-R2	ACCTSTTAATACTGCATTTR-CAT		
Anaplasmataceae	nested PCR	16S	Ana-F1	GAACGAACGCTGGCGG-CAAGC	500bp	[3]
			Ana-R1	AGTAYCGRACCAGA-TAGCCGC		
			Ana-F2	TGCATAGGAATCTACCTAG		
			Ana-R2	CTAGGAATTCCGCTATCCTCT		
A. marginale	semi-nested PCR	16S	A.mar-16s-F	GGATAGCCAC-TRGAARTGGTG	900bp	[2]
			A.mar-16s-R1	CGTGCTGACTTGACATCAT		
			A.mar-16s-R2	CATCTCACGACACGAGCTG		
	semi-nested PCR	*gltA*	A.mar-*gltA*-F1	TGGTAGAAAAAGCGAT-TTTAG	1200bp	
			A.mar-*gltA*-F2	ATAAGCTTGCCCGTTATGC		
			A.mar-*gltA*-R	CCGGTATAAAGTTGGCGT		
	semi-nested PCR	*groEL*	A.mar-*groEL*-F1	ACATGCTCCATACTGACTGC	860bp	
			A.mar-*groEL*-F2	AGATGAGATTGCACAGGTTG		
			A.mar-*groEL*-R	AGATGCAAGCGTG-TATAGCAG		
A．capra	nested PCR	16S	A.cap-16S-F1	TCCTGGCTCAGAACGAAC-GCTGGCG	1261bp	[4]
			A.cap-16S-R1	AGTCACTGACCCAAC-CTTAAATGGCTG		
			A.cap-16S-F2	GCAAGTCGAACGGAC-CAAATCTGT		
			A.cap-16S-R2	CCACGATTACTAGCGAT-TCCGACTTC		
	semi-nested PCR	*gltA*	A.cap-*gltA*-F1	ATGATCCGGGGTTCCTGTC	930bp	[5]
			A.cap-*gltA*-F2	TGCAGGTCTGAGATAACCT		
			A.cap-*gltA*-R	TACAATACCGGAGTAAAAGT		

	PCR	*groEL*	A.cap-*groEL* -F	TGAAGAG-CATCAAACCCGAAG	874 bp	[4]
			A.cap-*groEL* -R	CTRCTCGTGATGCTATCGG		
*A.platys&Ca.*A.cinen-sis	nested PCR	16S	A.pla-16s-F1	TCCTGGCTCAGAACGAAC-GCTGGCG	900bp	[6]
			A.pla-16s-R1	AGTCACTGACCCAAC-CTTAAATGGCTG		
			A.pla-16s-F2	GTCGAACGGAT-TATTCTTTATAGCTTGC		
			A.pla-16s-R2	CCCTTCCGTTAA-GAAGGATCTAATCTCC		
	semi-nested PCR	*gltA*	A.pla-*gltA*-F1	TGRAAGAAAAWGCTGTTTTG	870bp	[7]
			A.pla-*gltA*-F2	AGCTRTTTTRGAGTGYGGAG		
			A.pla-*gltA*-R	GCTCTRGGRTCATARCTYTT		
	semi-nested PCR	*groEL*	A.pla-*groEL* -F1	AGTCGATTAGGGAAGTAG-TAC	1000bp	
			A.pla-*groEL* -F2	AGGATGGCTACAAGGTAATG		
			A.pla-*groEL* -R	GCGTCCTCTACTCTGTCTT		
Ehrlichia	semi-nested PCR	16S	E-rrs1-F	GAATAGCCATT-AGAAATGATG	500bp	[2]
			E-rrs1-R1	GTCAGTATCGAACCAGATAG		
			E-rrs1-R2	GTATCGAACCAGATAGCCG		
	semi-nested PCR	16S	E-rrs2-F1	CGGCTATCTGGTTCGATAC	750bp	
			E-rrs2-F2	CTATCTGGTTCGATACTGAC		
			E-rrs2-R	GCTTCCTTKCGGTTAGCAC		
	semi-nested PCR	*gltA*	E-*gltA*-F1	CAG-GHTTTATGTCWACTGCTGCT	1000 bp	[8]
			E-*gltA*-F2	TTATGTCWACTGCTGCTT-GTGA		
			E-*gltA*-R	TAYAAYTGACGWGGAC-GACAT		
	semi-nested PCR	*groEL*	E-*groEL*-F1	TGGGCTGGYAATGAAATTGA	1100bp	[9]
			E-*groEL*-F2	AACATGGCAAATGTAGTTGT		
			E-*groEL*-R	TCAACAGCAGCTCTAGTTG		

Table S3. Statistical table of developmental stage and blood-soaked state of ticks.

	Life Stage			Blood-Soaked State	
	**Larval**	**Nymph**	**Adult**	**Half-Full Blood**	**Full Blood**
The number of ticks	0	97	295	257	135

Table S4. The positive rate and quantity of Rickettsiales bacteria detected in this study.

Rickettsia		***Anaplasma***				***Ehrlichia***	
Ca. R. jingxinensis	*R. japonica*	*A. platys*	*Ca.* A. cinensis	*A. marginale*	*A. capra*	*Ehrlichia sp.*	*E. minasensis*
15(3.8%)	36(9.2%)	2(0.5%)	2(0.5%)	46(11.7%)	2(0.5%)	64(16.3%)	6(1.5%)

References

1. Cao, W.C.; Zhao, Q.M.; Zhang, P.H.; Yang, H.; Wu, X.M.; Wen, B.H.; Zhang, X.T.; Habbema, J.D. Prevalence of *Anaplasma phagocytophila* and *Borrelia burgdorferi* in *Ixodes persulcatus* ticks from northeastern China. *The American journal of tropical medicine and hygiene* 68 **2003**: 547-550. 10.4269/ajtmh.2003.68.547.
2. Guo, W.P.; Tian, J.H.; Lin, X.D.; Ni, X.B.; Chen, X.P.; Liao, Y.; Yang, S.Y.; Dumler, J.S.; Holmes, E.C.; Zhang, Y.Z. Extensive genetic diversity of Rickettsiales bacteria in multiple mosquito species. *Scientific reports* 6 **2016**: 38770. 10.1038/srep38770.
3. Jafar Bekloo, A.; Ramzgouyan, M.R.; Shirian, S.; Faghihi, F.; Bakhshi, H.; Naseri, F.; Sedaghat, M.; Telmadarraiy, Z. Molecular Characterization and Phylogenetic Analysis of *Anaplasma* spp. and *Ehrlichia* spp. Isolated from Various Ticks in Southeastern and Northwestern Regions of Iran. *Vector Borne and Zoonotic Diseases (Larchmont, N.Y.)* 18 **2018**: 252-257. 10.1089/vbz.2017.2219.
4. Yang, J.; Liu, Z.; Niu, Q.; Liu, J.; Han, R.; Guan, G.; Hassan, M.A.; Liu, G.; Luo, J.; Yin, H. A novel zoonotic *Anaplasma* species is prevalent in small ruminants: potential public health implications. *Parasites & Vectors* 10 **2017**: 264. 10.1186/s13071-017-2182-9.
5. Guo, W.P.; Zhang, B.; Wang, Y.H.; Xu, G.; Wang, X.; Ni, X.; Zhou, E.M. Molecular identification and characterization of *Anaplasma capra* and *Anaplasma platys*-like in *Rhipicephalus microplus* in Ankang, Northwest China. *BMC infectious diseases* 19 **2019**: 434. 10.1186/s12879-019-4075-3.
6. Barlough, J.E.; Madigan, J.E.; DeRock, E.; Bigornia, L. Nested polymerase chain reaction for detection of *Ehrlichia* equi genomic DNA in horses and ticks (*Ixodes pacificus*). *Veterinary parasitology* 63 **1996**: 319-329. 10.1016/0304-4017(95)00904-3.
7. Guo, W.P.; Tie, W.F.; Meng, S.; Li, D.; Wang, J.L.; Du, L.Y.; Xie, G.C. Extensive genetic diversity of *Anaplasma bovis* in ruminants in Xi'an, China. *Ticks Tick Borne Dis* 11 **2020**: 101477. 10.1016/j.ttbdis.2020.101477.
8. Loftis, A.D.; Ross, D.E.; Levin, M.L. Susceptibility of mice (Mus musculus) to repeated infestation with *Amblyomma americanum* (Acari: Ixodidae) ticks. *Journal of medical entomology* 41 **2004**: 1171-1174, doi:10.1603/0022-2585-41.6.1171.
9. Lu, M.; Meng, C.; Gao, X.; Sun, Y.; Zhang, J.; Tang, G.; Li, Y.; Li, M.; Zhou, G.; Wang, W., et al. Diversity of Rickettsiales in *Rhipicephalus microplus* Ticks Collected in Domestic Ruminants in Guizhou Province, China. *Pathogens (Basel, Switzerland)* 11 **2022**:10.3390/pathogens11101108.
